# Severe hypothyroidism presenting as reversible proteinuria: two case reports

**DOI:** 10.1186/s13256-019-2216-3

**Published:** 2019-08-28

**Authors:** Ranga Migara Weerakkody, Pushpa Nandani Lokuliyana

**Affiliations:** 1 Department of Nephrology, Teaching Hospital, Jaffna, Sri Lanka; 2 Department of Pain Medicine, National Hospital of Sri Lanka, Regent Street, Colombo, 10 Sri Lanka

**Keywords:** Hypothyroidism, Proteinuria, Rhabdomyolysis, Acute renal failure, Creatinine kinase

## Abstract

**Background:**

Hypothyroidism is a common medical disorder which results in many metabolic effects, such as dyslipidemia, hypertension, accelerated atherosclerosis, and coronary artery disease. Hypothyroidism affects the renal physiology by affecting the renal blood flow, vascular resistance, and sodium handling. Recent studies have shown hypothyroidism is associated with decreased estimated renal function and proteinuria. Rhabdomyolysis and acute renal insufficiency have also been described in association with hypothyroidism. The severity of the proteinuria was directly proportional to thyroid-stimulating hormone levels. Currently, there is a lack of evidence on the reversibility of proteinuria in hypothyroidism. This is the first report in the literature, to the best of our knowledge, demonstrating the reversibility of proteinuria associated with hypothyroidism, with treatment.

**Case presentation:**

Here we describe two cases, a 72-year-old Sinhalese man and a 47-year-old Tamil woman, from Sri Lanka, presenting with overt hypothyroidism; they were found to have elevated creatinine, proteinuria, and elevated creatinine kinase levels. Due to lack of active sediment in urine analysis, these patients were observed after the initiation of thyroxine therapy. They were investigated in the adult-onset proteinuria pathway, excluding common reasons for proteinuria. Both patients responded to treatment: their serum creatinine, creatinine kinase, and urine protein levels reverted to physiological levels within 6 months of treatment with thyroxine, and with normalization of thyroid-stimulating hormone.

**Conclusion:**

Hypothyroidism can present as renal insufficiency, proteinuria, and can mimic rhabdomyolysis. Prompt initiation of thyroxine treatment and control of thyroid-stimulating hormone levels could reverse these changes.

## Background

Hypothyroidism is a common illness in medical practice and is subdivided into overt and subclinical varieties. Overt hypothyroidism (OHT) has been recognized for many years; OHT is defined as elevated serum thyroid-stimulating hormone (TSH) levels with reduced free thyroxine (FT) levels. Recently, an entity called subclinical hypothyroidism (SCHT) was described. SCHT is defined as elevated serum TSH levels with normal FT levels [1]. Several studies showed that SCHT is associated with dyslipidemia, hypertension, accelerated atherosclerosis, and coronary artery disease. Even a slight elevation in TSH levels has been shown to be associated with accelerated atherosclerosis [2–4]; hence, it is an important clinical entity. Many experimental studies assessed vascular resistance, renal sodium handling, and measured renal blood flow in hypothyroidism, and most described significant changes from the physiological state [5]. A recent study by Chang *et al.* looked at the glomerular filtration rate and proteinuria in patients with hypothyroidism [5]. The above study demonstrated that there is significant difference in proteinuria in OHT and SCHT compared to the normal population, and the severity of proteinuria is associated with TSH levels. Many studies claimed that estimated glomerular filtration rate drops in hypothyroidism [6]. In addition, rhabdomyolysis and acute renal failure [7–9] were described with severe hypothyroidism.

We describe two cases of severe hypothyroidism presenting with elevated serum creatinine and proteinuria, which normalized on treatment with thyroxine. A renal biopsy is an expensive investigation and has complications; it is the ultimate diagnostic tool in proteinuria. This case report helps to show that at least in hypothyroidism, a clinician could delay a renal biopsy until thyroid function normalizes.

## Case presentation

### Case 1

A 72-year-old Sinhalese man, a paddy farmer from Western Province, Sri Lanka, presented with complaints of facial puffiness and body aches during exertion. He was a healthy man with no history of long-term medications, he did not consume alcohol, and he did not smoke tobacco. On further questioning he complained of cold intolerance; he had no frothy urine and no features of a connective tissue or autoimmune disorder. He had good exercise tolerance and had never experienced ischemic-type chest pains. We excluded history of recent trauma or seizures by careful detailed questioning. He has no family history of renal disease. He was from a rural area of the Western Province, with access to clean water and sanitation. He gave a history of exposure to various pesticides and weedicides that he has used for nearly 45 years as a farmer. On examination a hoarse voice was noted, with puffy swelling of his body. A mild pallor was noted on examination. His blood pressure was 117/74 mmHg and pulse rate was 62/minute. Other than for sluggishness of reflexes, a neurological examination was unremarkable. A clinical diagnosis of hypothyroidism was made and he was followed up with blood investigations. A TSH > 100 U/L confirmed the diagnosis. In addition, a serum creatinine of 167 umol/L was noted with a urine analysis showing 250 mg/dL albuminuria, and blood urea of 4.6 mmol/L. His urine protein to creatinine ratio (UPCR) was 3.4. He had elevated lipid levels. An extremely low blood urea to creatinine ratio prompted us to exclude coexisting liver disease or myopathy. Liver function tests were normal, but creatinine kinase (CK) was grossly elevated to 4473 U/L. A normal 9.00 a.m. cortisol level ruled out coexisting hypoadrenalism. He was started on an escalating dose of thyroxine, starting with 25 μg daily, with 25 μg increments every fortnight, up to 100 μg/day. Hepatitis B, hepatitis C, and HIV serology were negative. His erythrocyte sedimentation rate (ESR) was 25, and serum protein electrophoresis was normal. An ultrasound scan of his abdomen revealed normal-looking kidneys and did not demonstrate any lymphadenopathy. Antinuclear factor, C3 level, and C4 level were unremarkable. A renal biopsy was not performed initially as rhabdomyolysis was not a likely diagnosis, and was not performed later due to rapid resolution with thyroxine. An ultrasound scan plus duplex of his thyroid revealed a multinodular goiter with no prominent or vascular nodules.

He was followed up at 2, 4, and 6 months. His proteinuria disappeared by 16 weeks, creatinine gradually dropped down to 88 umol/L, and CK normalized to 125 U/L. TSH at 6 months was 1.20 U/L. Omega 3 fatty acids were started to counter the hyperlipidemia, and was converted at 4 months to rosuvastatin 5 mg daily, which was omitted at 6 months.

### Case 2

A 47-year-old Tamil woman from Northern Sri Lanka was referred by a peripheral clinic for further evaluation of elevated serum creatinine. She had been hypertensive for 5 years; she did not have a history of diabetes or ischemic heart disease. She was on treatment for hypertension and hypercholesterolemia with enalapril 5 mg daily and fenofibrate 200 mg daily. She did not have a history suggestive of renal disease, autoimmune disorder, or connective tissue disorder. She failed to recall any history of major trauma, dehydration, ingestion of drugs and/or toxins, or seizures within the last few weeks. She is a housewife and mother of two children. Similar to many Asian women, she did not consume alcohol and she did not smoke tobacco. She was married to a hospital clerk, and did not recall exposure to toxins. She was not living in an endemic area of chronic interstitial nephritis in agricultural communities (CINAC).

She had a blood pressure of 150/100 mmHg, with normal cardiovascular examination. She was not pale. She did not have any edema on examination. Her serum creatinine was 126 umol/L, with a blood urea of 3.2 mmol/L. Urine analysis revealed bland sediment with 100 mg/dL of protein, but no hemoglobin or myoglobin. A full blood count showed hemoglobin of 112 g/L, with a mean corpuscular volume of 98 fl. This raised the possibility of hypothyroidism. Further investigations showed a UPCR of 1.6, elevated serum lipids, TSH of > 100 U/L, and CK of 3980 U/L. Her liver profile showed alanine transferase (ALT) of 45 (reference range < 30), aspartate transferase (AST) of 56 (reference range < 30), and alkaline phosphatase (ALP) of 122 (reference range < 245), slight derangement. An initial diagnosis of fenofibrate-induced rhabdomyolysis was made, and fenofibrate was withdrawn from the treatment. She was initiated on management of rhabdomyolysis with alkaline diuresis. An ultrasound scan of her abdomen revealed normal-looking kidneys and no lymphadenopathy. Hepatitis B, hepatitis C, HIV, antinuclear factor, C3 level, and C4 level, were all within reference ranges. Urine myoglobin and urine hemosiderin deposits were negative. However, there was no change in her CK levels (3870 U/L) or creatinine (133 umol/L) levels after a lapse of 2 weeks, and we decided her elevated CK levels were unlikely to be due to fenofibrate-induced rhabdomyolysis. We assumed it was due to hypothyroidism and started an escalating dose of thyroxine at 25 μg/day, with increments of 25 μg each 2 weeks, up to 75 μg/day. A renal biopsy was not performed initially as rhabdomyolysis was not likely, and it was not performed later due to rapid resolution with thyroxine. An ultrasound plus duplex of her thyroid revealed a multinodular goiter with no prominent or vascular nodules. Her CK gradually dropped over the next 12 weeks, with her creatinine, to 93 umol/L. Her UPCR reduced to 0.6 after 6 months of treatment. At the end of 6 months of follow-up her renal function and thyroid functions normalized, and proteinuria was absent.

**Fig. 1:**
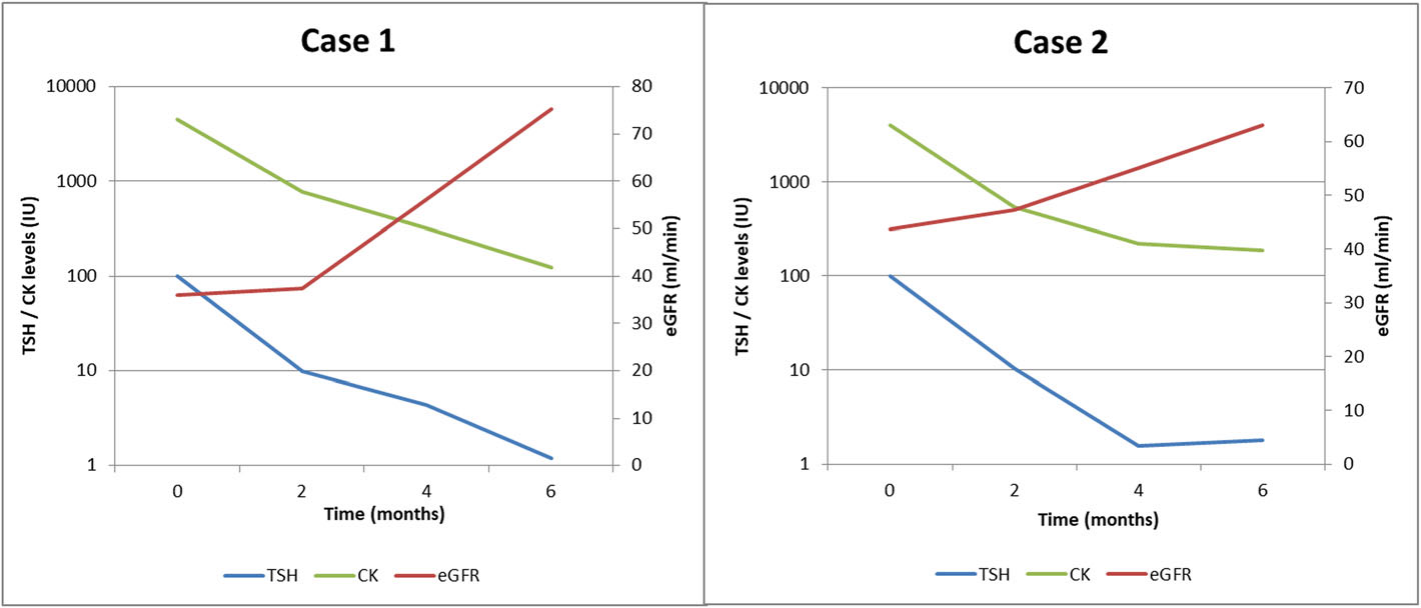
The changes of throid stimulating hormone, creatinine kinase and estimated glomerular filtration rates of the two patient during study period

In both patients, thyroglobulin antibodies tests were not performed due to economic constraints. A summary of the investigations is given in Table 1. Figure 1 gives a graphical representation of change of renal functions and CK levels with TSH.

**Table 1:** Summary of investigations

	Reference range	Case 1 – follow-up (months)				Case 2 – follow-up (months)			
		0	2	4	6	0	2	4	6
TSH	0.4–4.0 U/L	100	9.8	4.4	1.2	100	10.42	1.56	1.8
Free T4	7–19 ng/L	0.2	7.9	8.6	9.1	0.3	6.1	7.7	10.2
Creatinine	44–105 umol/L	167	157	112	88	126	118	104	93
eGFR	> 90 ml/minute	36	37.4	56.2	75.3	43.7	47.3	55.1	63
Blood urea	2.5–7.1 mmol/L	4.6	4.8	5	5.2	3.2	3.3	3.3	4
CK	22–198 U/L	4473	765	322	125	3980	534	221	189
UPCR	< 0.4 mg/mg	3.2	2.4	0.4	0.4	1.6	0.9	0.7	0.6
Cholesterol	< 200 mg/dL	622	417	221	197	478	332	225	172
Triglycerides	< 150 mg/dL	254	156	102	93	332	165	83	92
Hemoglobin	110–145 g/L	102	105	118	131	112	117	119	121
MCV	80–90 fL	102	99	96	92	97	95	92	89
HbA1c	< 6.0%	5.90%				5.20%			

*CK* creatinine kinase, *eGFR* estimated glomerular filtration rate, *HbA1c* glycated hemoglobin, *MCV* mean corpuscular volume, *T4* thyroxine, *TSH* thyroid-stimulating hormone, *UPCR* urine protein to creatinine ratio

## Discussion

Hypothyroidism is a common medical disorder, which is defined by elevated TSH levels above 4.0 mU/L. Depending on the free triiodothyronine and thyroxine levels; it is further subdivided into OHT or SCHT. The description of proteinuria in hypothyroidism is a novel finding [5], and its reversibility has not been studied before. This is a report of two cases that describes the reversibility of proteinuria in hypothyroidism with appropriate treatment.

Hypothyroidism has many metabolic implications. Its effect on renal physiology, including vascular resistance, renal sodium handling, and renal blood flow, has been described as producing significant changes from the physiological state [5]. Many studies showed an association between hypothyroidism and elevated serum creatinine, most probably due to myopathy, and the reversal of it with thyroxine treatment [7–9]. The studies that looked at renal functions in hypothyroidism used estimated glomerular filtration rate instead of measured glomerular filtration rate, using inulin or iothalamate [5, 10]. The estimation equations cannot be used in the presence of elevated CK levels (indicating muscle injury); hence, the claims of acute kidney injury or impaired renal functions in hypothyroidism need to be treated with some care. The normal blood urea levels of our two patients, which only changed slightly over the duration of treatment, support the claim of absence of acute kidney injury.

The reasons for myopathy are unclear, but changes in intramuscular glycogen metabolism and changes of physiology of mitochondria have been proposed [8]. In both these patients we excluded causes of rhabdomyolysis. In the first case, it was a straightforward history-taking exercise, with absence of any risk factors. However, in the second case, our patient was on fenofibrate, which is a well-recognized factor for rhabdomyolysis. The failure of CK to respond to fenofibrate withdrawal and the near normal liver functions tests ruled out (fibrate induced) rhabdomyolysis. The CK levels were typically lower than a case of rhabdomyolysis as well.

A recent study by Chang *et al.* showed significant proteinuria associated with hypothyroidism [5], the severity increasing with that of hypothyroidism. However, very few studies reported normalization of elevated CK and proteinuria with thyroxine treatment. Hypothyroidism has been found to be associated with glomerular pathologies, such as membranous glomerulopathy, minimal change disease, and membranoproliferative glomerulonephritis. However, the authors were not able to find a plausible explanation for the development of proteinuria in hypothyroidism, even after extensive reference to the literature [11–14]. Our patients only had bland urine sediment and nephrotic range proteinuria. The inflammatory markers, C3/C4 and antinuclear antibodies (ANA) were normal. There were no features to suggest advanced glomerulopathies. Renal biopsies were obviously not performed due to lack of benefit, as these patients rapidly responded to thyroxine. In both patients, other common causes for adult-onset proteinuria had been excluded with common infections, systematic lupus erythematous, or plasma cell dyscrasias (in first patient). It is prudent to think that the proteinuria was a result of hypothyroidism, because of a lack of other causative factors and rapid resolution on treatment with thyroxine. Measurements of thyroglobulin antibody titers would have given additional information, but our patients could not afford it due to economic constraints.

## Conclusions

Severe hypothyroidism can present as renal insufficiency and proteinuria. Prompt initiation of thyroxine treatment and control of TSH levels could reverse these changes.

## Data Availability

Data sharing is not applicable to this article as no datasets were generated or analyzed during the current study.

## Abbreviations

ALP: Alkaline phosphatase
ALT: Alanine transferase
ANA: Antinuclear antibodies
AST: Aspartate transferase
CINAC: Chronic interstitial nephritis in agricultural communities
CK: Creatinine kinase
ESR: Erythrocyte sedimentation rate
FT: Free thyroxine
OHT: Overt hypothyroidism
SCHT: Subclinical hypothyroidism
TSH: Thyroid-stimulating hormone
UPCR: Urine protein to creatinine ratio
